# Joint neutron/X-ray crystal structure of a mechanistically relevant complex of perdeuterated urate oxidase and simulations provide insight into the hydration step of catalysis

**DOI:** 10.1107/S2052252520013615

**Published:** 2021-01-01

**Authors:** Lindsay McGregor, Tamás Földes, Soi Bui, Martine Moulin, Nicolas Coquelle, Matthew P. Blakeley, Edina Rosta, Roberto A. Steiner

**Affiliations:** a Randall Centre for Cell and Molecular Biophysics, King’s College London, London SE1 1UL, United Kingdom; b Large Scale Structures Group, Institut Laue-Langevin, 71 avenue des Martyrs, 38042 Cedex 9, Grenoble, France; c Department of Chemistry, King’s College London, London SE1 1DB, United Kingdom; d Department of Physics and Astronomy, University College, London WC1E 6BT, United Kingdom; e Life Sciences Group, Institut Laue-Langevin, 71 avenue des Martyrs, 38042 Cedex 9, Grenoble, France

**Keywords:** neutron/X-ray diffraction, urate oxidase, protein perdeuteration, cofactor-independent oxidase, biomolecular simulations

## Abstract

Cofactor-independent urate oxidase (UOX) is an ∼137 kDa tetrameric enzyme essential for uric acid (UA) catabolism in many organisms. UA is first oxidized by O_2_ to de­hydro­isourate (DHU) via a peroxo intermediate. DHU then undergoes hydration to 5-hy­droxy­isourate (5HIU). At different stages of the reaction both catalytic O_2_ and water occupy the ‘peroxo hole’ above the organic substrate. Here, high-resolution neutron/X-ray crystallographic analysis at room temperature has been integrated with molecular dynamics simulations to investigate the hydration step of the reaction. The joint neutron/X-ray structure of perdeuterated *Aspergillus flavus* UOX in complex with its 8-azaxanthine (8AZA) inhibitor shows that the catalytic water molecule (W1) is present in the peroxo hole as neutral H_2_O, oriented at 45° with respect to the ligand. It is stabilized by Thr57 and Asn254 on different UOX protomers as well as by an O—H⋯π interaction with 8AZA. The active site Lys10–Thr57 dyad features a charged Lys10–NH_3_^+^ side chain engaged in a strong hydrogen bond with Thr57^OG1^, while the Thr57^OG1–HG1^ bond is rotationally dynamic and oriented toward the π system of the ligand, on average. Our analysis offers support for a mechanism in which W1 performs a nucleophilic attack on DHU^C5^ with Thr57^HG1^ central to a Lys10-assisted proton-relay system. Room-temperature crystallography and simulations also reveal conformational heterogeneity for Asn254 that modulates W1 stability in the peroxo hole. This is proposed to be an active mechanism to facilitate W1/O_2_ exchange during catalysis.

## Abstract 2

The joint neutron/X-ray crystallographic structure of perdeuterated *Aspergillus flavus* urate oxidase in complex with the inhibitor 8-azaxanthine and a catalytic water bound in the peroxo hole is presented together with an integrated analysis using biomolecular simulations.

## 1. Introduction   

In many organisms, urate oxidase (UOX) is an essential enzyme that catalyses the O_2_-dependent degradation of uric acid (UA) to 5-hy­droxy­isourate (5HIU) (Kahn *et al.*, 1997 ▸). Humans, however, lack UOX, as it was lost by pseudogenization during primate evolution resulting in inactivation of the uricolytic pathway (Oda *et al.*, 2002 ▸). Therefore, recombinant UOX finds a therapeutic application for the enzymatic treatment of severe hyperuricemia and prevents the UA burst accompanying tumour lysis after certain chemotherapy treatments (Terkeltaub, 2010 ▸).

**Figure 1:**
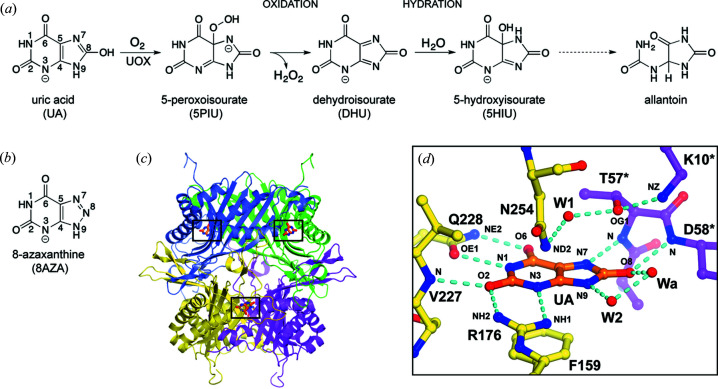
UOX catalysis. (*a*) Reaction scheme for UOX catalysis highlighting the separate oxidation and hydration steps. UOX catalyses the transformation of UA to 5-HIU. The latter is then converted to allantoin either enzymatically or non-enzymatically. (*b*) Chemical structure of the inhibitor 8-azaxanthine (8AZA) used in this study. (*c*) Cartoon representation of the ∼137 kDa UOX tetramer with the four protomers highlighted by different colours. Bound ligands are shown as orange sticks with their active site location marked by black rectangles. UOX binds four ligands at independent active sites each contributed by two UOX protomers. Only three are visible in the current view. (*d*) Stick representation of UA (orange) bound in the active site at the interface between two different UOX protomers, shown in yellow and magenta, respectively. Nitro­gen and oxygen atoms are in blue and red, respectively. Water molecules (W1, W2, Wa) in close proximity to UA are represented by spheres. Hydrogen bonds are shown by cyan broken lines. Residues labelled with an asterisk indicate that they belong to a different UOX protomer. Panels (*c*) and (*d*) were generated using coordinates from the anaerobic UOX–UA complex (PDB entry 4d12; Bui *et al.*, 2014 ▸). Chemical and molecular graphics representations in this work were produced with *CHEMDRAW* (Perkin Elmer) and *PyMOL* (Schrödinger, LLC) (DeLano, 2002 ▸), respectively, and assembled using *Adobe Illustrator* (Adobe).

Mechanistically, UOX-mediated degradation of UA follows two sequential steps: (1) an initial oxidation step whereby UA reacts with O_2_ to yield de­hydro­isourate (DHU) *via* a 5-peroxoisourate (5PIU) intermediate and (2) a hydration step in which DHU is hy­droxy­lated to 5HIU [Fig. 1 ▸(*a*)] (Kahn, 1999 ▸; Wei *et al.*, 2017 ▸). The latter is ultimately transformed to the more soluble allantoin either enzymatically or non-enzymatically (Ramazzina *et al.*, 2006 ▸). High-resolution X-ray crystallographic studies of the anaerobic complex of *Aspergillus flavus* UOX (hereafter simply UOX) in complex with its natural substrate UA (Bui *et al.*, 2014 ▸) as well as with several other similar scaffolds including the 8-azaxanthine (8AZA) inhibitor employed in this study [Fig. 1 ▸(*b*)] (Colloc’h *et al.*, 1997 ▸; Gabison *et al.*, 2010 ▸; Retailleau *et al.*, 2004 ▸), reveal that ligands bind at the interface between two protomers of the ∼137 kDa UOX tetramer stabilized by several hydrogen bonds with the additional contribution of a π–π stacking interaction [Figs. 1 ▸(*c*) and 1(*d*)]. Directly above the ligand plane, a water molecule (W1) suggested to be responsible for the hydration step of catalysis (Wei *et al.*, 2017 ▸) is coordinated by the side chains of Thr57* and Asn254 (an asterisk indicates residues belonging to the second UOX molecule contributing to the active site). A comparison with the X-ray structure of UOX in complex with the 5PIU intermediate trapped under cryo-conditions (Bui *et al.*, 2014 ▸) shows that W1 occupies the same position of the peroxo group [Fig. S1(*a*) of the supporting information]. This location (the ‘peroxo hole’) also provides stabilization to O_2_ during the formation of the 5PIU intermediate (Bui *et al.*, 2014 ▸) [Fig. S1(*b*)]. Small negative ions like cyanide and chloride have been visualized in the peroxo hole in the presence of intact UA under aerobic conditions (Gabison *et al.*, 2008 ▸, 2010 ▸; Oksanen *et al.*, 2014 ▸) offering structural support for their behaviour as competitive inhibitors.

UOX belongs to a group of intriguing cofactor-independent oxidases and oxygenases that rely on their organic substrates for O_2_ activation (Fetzner & Steiner, 2010 ▸). At optimal catalytic pH (∼8.0) (Pfrimer *et al.*, 2010 ▸), UA is a monoanion in solution and an early mechanistic proposal suggested the existence of a UOX general base dyad formed by Lys10* and Thr57* which further deprotonate UA upon binding, generating the dianion (Imhoff *et al.*, 2003 ▸). A signature for the latter has been identified spectroscopically during catalysis (Kahn & Tipton, 1998 ▸). As deduced by its tendency to undergo spontaneous oxidation in the gas phase (Kahn & Tipton, 1997 ▸), the UA dianion has been proposed to transfer one electron to O_2_ to generate the superoxide anion (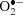). This would result in a [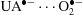] pair that, following radical recombination, yields 5PIU. However, a recent theor­etical study questioned the involvement of the UA dianion (Wei *et al.*, 2017 ▸). Instead, it proposed a reaction path in which the Lys10*–Thr57* dyad abstracts the H8 proton from the UA monoanion bound in its C8-lactim form [tautomer shown in Fig. 1 ▸(*a*)] with a concomitant one-electron transfer to O_2_ resulting directly in the formation of the [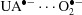] pair. The latter mechanism is supported by the neutron crystallographic structure of the UOX–UA complex solved under aerobic conditions in the presence of inhibitory chloride in the peroxo hole (UOX–UA–Cl^−^, PDB entry 4n9m; Oksanen *et al.*, 2014 ▸) which revealed UA bound in its monoanion form deprotonated at N3 with a deuteron bound to O8. Deproton­ation at N3 was also observed for 8AZA in the neutron structure of the UOX–8AZA–Cl^−^ complex (PDB entry 4n3m; Oksanen *et al.*, 2014 ▸).

Displacement of W1 by chloride in the neutron structures currently available precludes obtaining direct information on the catalytic water and on the protonation state of its environment relevant to the hydration step of catalysis. Here, we have integrated high-resolution room-temperature neutron/X-ray crystallographic structural analysis with molecular dynamics (MD) simulations to study the UOX–8AZA complex in the presence of W1 in the peroxo hole. To maximize the signal-to-noise ratio (S/N) of the neutron diffraction data we employed perdeuterated UOX (^D^UOX) instead of relying on protium (^1^H)/deuterium (^2^H or D) replacement of exchangeable hydrogens as done in previous studies on this system (Oksanen *et al.*, 2014 ▸). Protein perdeuteration, which replaces all hydrogen atoms with deuterium, is an effective method of improving S/N as it dramatically lowers the incoherent background while enhancing the coherent scattering signal (Fisher *et al.*, 2014 ▸; Shu *et al.*, 2000 ▸). In addition, perdeuteration avoids map cancellation issues, allowing all D atoms to be visualized at ∼2.5 Å resolution or better (Blakeley & Podjarny, 2018 ▸; Logan, 2020 ▸).

The high-resolution joint neutron/X-ray structure of ^D^UOX–8AZA–W1 presented here provides experimental support for a mechanism in which W1 attacks DHU^C5^ (atom C5 of DHU) with the assistance of the Lys10*–NH_3_^+^⋯HO–Thr57* dyad in which Thr57*^HG1^ is central to a proton-relay system. Experiment and simulations highlight a degree of rotational freedom for the Thr57* hydroxyl group. We suggest that this helps Thr57* to fulfil a dual role of stabilizing W1 as well as acting as a proton donor to the DHU^N7^ π system to kickstart hy­droxy­lation. Moreover, experiment and simulations also reveal an unforeseen conformational heterogeneity for Asn254 that modulates W1 stability in the peroxo hole. This might represent an active mechanism that facilitates W1/O_2_ exchange during catalysis.

## 2. Materials and methods   

### 2.1. Perdeuterated *A. flavus* UOX (^D^UOX)   

*A. flavus* urate oxidase (UOX) cDNA codon-optimized for bacterial expression was purchased from Genscript (Piscataway, NJ, USA) and cloned using the NdeI/XhoI restriction sites in a pET24b(+) expression vector (Novagen). Recombinant untagged perdeuterated UOX (^D^UOX) was expressed in *E. coli* BL21 (DE3) cells. Cells were adapted for growth under deuterated conditions via a stepwise adaptation process using a minimal medium with glycerol-d_8_ as the carbon source. ^D^UOX was produced in a large quantity via batch-fed fermentation (Haertlein *et al.*, 2016 ▸). ^D^UOX was purified using a multi-step protocol previously developed for protiated UOX (Bui *et al.*, 2014 ▸). Briefly, pelleted cells were re-suspended in 50 m*M* Tris–HCl (pH 8.0), 250 m*M* NaCl, supplemented with lysozyme, DNAse and a protease inhibitor cocktail, and lysed by sonication. Protein purification was performed using a combination of ammonium sulfate precipitation, DEAE and Resource Q ion exchange, Phenyl Sepharose hydro­phobic interaction, and Superdex 75 size-exclusion chromatographic steps. After each step samples were analysed by SDS-PAGE and the purest fractions were pooled for further work. Both hydro­phobic interaction and size-exclusion purification steps were carried out in the absence of NaCl in the buffers.

### 2.2. Mass spectrometry   

Liquid chromatography electrospray ionization mass spectrometry (LC/ESI-MS) was performed on a 6210 LC-TOF spectrometer coupled to an HPLC system (Agilent Technologies). All solvents used were HPLC grade (Chromasolv, Sigma–Aldrich), tri­fluoro­acetic acid (TFA) was from Acros Organics (puriss p.a.). Solvent A was 0.03% TFA in water, solvent B was 95% aceto­nitrile, 5% water and 0.03% TFA. Just before analysis protein samples were diluted under acidic denaturing conditions to a final concentration of 5 µ*M* with solution A (0.03% TFA in water). Protein samples were firstly desalted on a reverse-phase C8 cartridge (Zorbax 300SB-C8, 5 µm, 300 µm ID × 5 mm, Agilent Technologies) for 3 min at a flow rate of 50 µl min^−1^ with 100% solvent A and then eluted with 70% solvent B at flow rate of 50 µl min^−1^ for MS detection. MS acquisition was carried out in positive ion mode in the 300–3200 *m*/*z* range. MS spectra were acquired and the data processed with the *MassHunter* workstation software (v.B.02.00, Agilent Technologies).

### 2.3. Crystallization, neutron/X-ray data collection and structural analysis   

^D^UOX eluted from size-exclusion chromatography in 50 m*M* Tris–acetate, 30 m*M* sodium acetate at pH 8.0 was thoroughly exchanged against 50 m*M* Tris–acetate, 30 m*M* sodium acetate in D_2_O, pD 7.59, before concentrating to >20 mg ml^−1^. ^D^UOX–8AZA crystals were grown at 291 K using the batch crystallization method. Deuterated crystallization buffer consisted of 50 m*M* Tris–acetate, 8% PEG 8000, 2 mg ml^−1^ 8AZA, pD 7.59. Crystallization drops of 30 µl total volume were prepared with protein solution and crystallization buffer in a 2:1 ratio. After ∼10 d, a single crystal of approximate volume 1.5 mm^3^ (1.4 × 1.2 × 0.9 mm) was mounted in a quartz capillary and sealed with wax for data collection.

Neutron quasi-Laue diffraction data to 2.10 Å resolution were collected at 298 K on the LADI-III instrument at the Institut Laue-Langevin (ILL), Grenoble, France (Blakeley *et al.*, 2010 ▸). Data were collected using the wavelength range 3.12–4.20 Å, centred at 3.68 Å, and an exposure time of 1.5 h per image. Neutron diffraction data were indexed and integrated with the program *LAUEGEN* (Campbell, 1995 ▸). *LSCALE* (Arzt *et al.*, 1999 ▸) was used in the wavelength normalization step, followed by data scaling and merging using *SCALA* (Evans, 2006 ▸), and conversion to structure factors using the program *TRUNCATE* (French & Wilson, 1978 ▸; Winn *et al.*, 2011 ▸). Neutron data collection statistics are given in Table 1 ▸. Subsequent to the neutron diffraction experiment, the same crystal was used for X-ray diffraction data collection at 298 K on the BM30A (FIP) beamline at the European Synchrotron Radiation Facility (ESRF), Grenoble, France. Data to 1.33 Å resolution were indexed and integrated using the *xia2* pipeline (Winter, 2010 ▸). Statistics for the X-ray dataset are also reported in Table 1 ▸.

Joint neutron/X-ray refinement was performed with the *phenix.refine* (Afonine *et al.*, 2010 ▸) package of the *Phenix* suite (Liebschner *et al.*, 2019 ▸) using the anisotropic treatment of ADPs for all non-hydrogen atoms. Deuterium atoms, added to the model at hydrogen positions using the *READYSET* program (Liebschner *et al.*, 2019 ▸) were refined isotropically. Model fitting to X-ray and neutron maps was carried out with the package *Coot* (Emsley *et al.*, 2010 ▸). Refinement statistics can be found in Table 2 ▸. The final model was deposited in the PDB (PDB entry 7a0l).

### 2.4. Molecular dynamics simulations   

Molecular dynamics (MD) simulations were performed with the *NAMD* 2.12 software (Phillips *et al.*, 2005 ▸, 2020 ▸). Parametrization of the system, including the 8AZA ligand, was performed through *CHARMM-GUI* (Jo *et al.*, 2008 ▸). The electrostatic description of the ligand was enhanced by ESP partial atomic charges, calculated separately with *Gaussian09* (Frisch *et al.*, 2013 ▸) at the B3LYP-BJD3/6-311G(d,p) level of theory (Becke, 1993 ▸; Grimme *et al.*, 2011 ▸; McLean & Chandler, 1980 ▸; Krishnan *et al.*, 1980 ▸) with the *CHELPG* algorithm (Breneman & Wiberg, 1990 ▸). The simulated systems were solvated in a TIP3P water box with the box size based on the dimensions of the system and giving 10 Å extension in all directions. Four K^+^ counterions were added to neutralize the system. For each system, our MD protocol consisted of the following steps: (1) energy minimization over 10 000 steps using a timestep of 2 fs; (2) equilibration over 1 ns in the NVT ensemble (*T* = 303.15 K) with the RMSD of heavy atoms of the host and guest molecules constrained to their initial position using a force constant of 1 kcal mol^−1^ Å^−2^; (3) production run in NPT ensemble with a time step of 2 fs, where the temperature and pressure were held at constant at 303.15 K and 1.0 atm, respectively. Constant temperature was set by a Langevin thermostat with a collision frequency of 1 ps^−1^, whereas the target pressure was reached using the Berendsen barostat. The *Shake* algorithm (Andersen, 1983 ▸) was used for bonds and angles involving hydrogen atoms. We used the particle mesh Ewald method (Darden *et al.*, 1993 ▸) for the long-range electrostatics in combination with a cutoff of 12 Å for the evaluation of the non-bonded interactions.

### 2.5. Quantum mechanics/molecular mechanics calculations   

Quantum mechanics/molecular mechanics (QM/MM) calculations, including geometry minimizations and molecular dynamics simulations, were performed with *CHARMM* molecular mechanics simulation software (Brooks *et al.*, 2009 ▸). Parametrization of the system was performed through *CHARMM-GUI* (Jo *et al.*, 2008 ▸). A cutoff distance of 12 Å was used for the Lennard–Jones and electrostatic interactions. In order to keep smooth transition to zero, shifting was applied to the pairwise interactions over 10 Å distances. Residues further than 25 Å from the ligand were deleted from the structures. We applied constraints on the atoms lying further than 15 Å from a ligand to keep them at their original positions. These geometry restrictions were defined for the starting structure and kept unaltered through the calculations. The forces acting on the QM region of the model were calculated externally with the *Q-Chem* 4.3 software suite (Shao *et al.*, 2015 ▸), which also involved the surrounding atoms as point charges. DFT calculations were performed at the B3LYP/6–31+G* level of theory. For geometry minimizations a limit of 1000 steps and a threshold for the RMSD of the gradient of 0.005 kcal mol^−1^ was applied. The connection between the QM and the MM regions was maintained with hydrogen link atoms.

## 3. Results   

### 3.1. Perdeuterated *A. flavus* UOX (^D^UOX) for neutron crystallography   

*A. flavus* UOX expressed recombinantly in *Saccharomyces cerevisiae* is a therapeutic commercial product of Sanofi–Aventis under the brand name Fasturtec. Several high-resolution structural studies on this enzyme relied on protein material kindly gifted by the company (Gabison *et al.*, 2010 ▸; Oksanen *et al.*, 2014 ▸; Retailleau *et al.*, 2004 ▸). To circumvent this limitation we previously set up a heterologous expression system in *E. coli* (Bui *et al.*, 2014 ▸). Using codon-optimized cDNA, untagged UOX can be expressed in good quantities and purified to homogeneity allowing us to obtain crystals that diffract X-rays to atomic resolution (Bui *et al.*, 2014 ▸). Bacterial expression of UOX from other organisms has also been reported enabling X-ray crystallographic studies to be performed, albeit at lower resolution (Hibi *et al.*, 2016 ▸; Marchetti *et al.*, 2016 ▸; Imhoff *et al.*, 2003 ▸).

**Figure 2:**
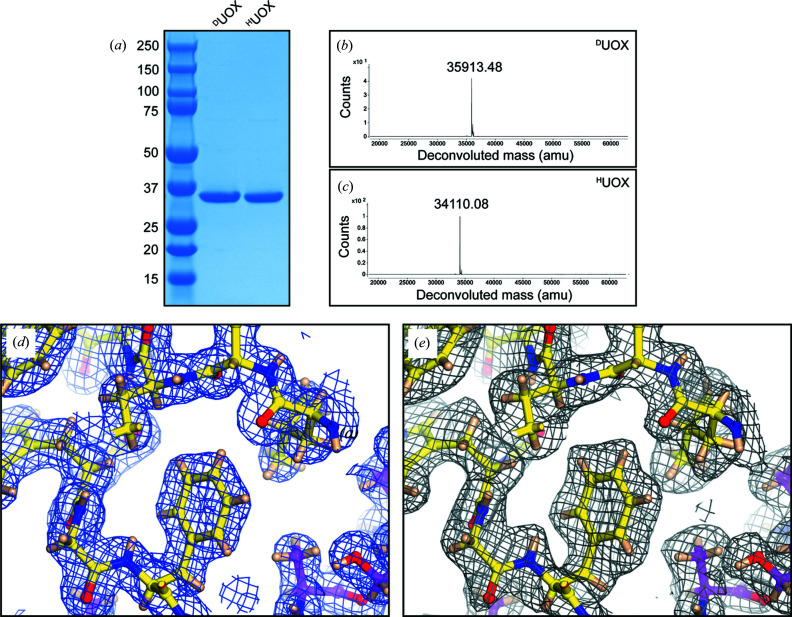
^D^UOX and examples of electron and neutron density maps for the ^D^UOX–8AZA–W1 complex. (*a*) SDS-PAGE analysis of purified ^D^UOX and ^H^UOX (protiated, for comparison) samples. Protein markers are on the left-hand side lane with their molecular weights indicated. (*b*), (*c*) ESI-MS analysis of purified (*b*) ^D^UOX and (*c*) ^H^UOX, showing a mass difference of 1803 a.m.u. As the calculated mass difference (assuming complete deuteration) is 1820 a.m.u., this corresponds to ∼99.1% deuterium incorporation in the ^D^UOX sample. (*d*), (*e*) Representative examples of 2*mF*_o_ − *DF*_c_ (*d*) electron density and (*e*) neutron maps contoured at the 1.0σ level. X-ray and neutron data extend to 1.33 and 2.10 Å, respectively. As in Fig. 1 ▸(*c*), residues shown in yellow and magenta belong to different UOX protomers. Nitro­gen, oxygen and deuterium atoms are shown in blue, red and wheat, respectively. The use of perdeuterated UOX provides neutron maps essentially free from cancellation effects affording clear visualization of both non-exchangeable and exchangeable hydrogens.

As neutron crystallographic studies benefit substantially from data collection on fully deuterated crystals we took advantage of the D-LAB facility (ILL, Grenoble, France) to produce perdeuterated UOX (^D^UOX) using our expression construct. Stepwise adaptation of *E. coli* to 100% D_2_O and the use of deuterated glycerol as a carbon source allowed us to obtain high levels of ^D^UOX after optimization of fermentation conditions (Haertlein *et al.*, 2016 ▸). Purification of ^D^UOX mirrored the strategy we established previously for (protiated) UOX affording more than 50 mg of pure ^D^UOX per litre of culture [Fig. 2 ▸(*a*)]. The degree of deuteration was estimated by MS at >99% [Figs. 2 ▸(*b*) and 2(*c*)].

### 3.2. Joint neutron/X-ray structure of the ^D^UOX–8AZA–W1 complex   

A single crystal (volume ≃ 1.5 mm^3^) of ^D^UOX in complex with 8AZA obtained under chloride-free conditions was utilized to collect sequentially room-temperature neutron and X-ray diffraction data at resolutions of 2.10 and 1.33 Å, respectively. Data collection statistics are given in Table 1 ▸. As the crystal was both perdeuterated and had a large volume, relatively short exposure times could be used for neutron data collection, allowing the complete dataset to be collected in only 16.5 h at the LADI-III beamline at the ILL (Blakeley *et al.*, 2010 ▸). Typically, neutron data collections take several days (Blakeley & Podjarny, 2018 ▸) and so the data collection for our study described here represents (as far as we are aware) the fastest neutron data collection for a protein with a relatively large unit cell. For X-ray data collection we did not attempt to measure data at the highest possible resolution. Instead, we opted to collect a complete dataset with negligible radiation damage. Electron density maps display none of the specific signatures typically associated with radiation damage, such as de­carboxyl­ation of acidic residues (Ravelli & McSweeney, 2000 ▸; Weik *et al.*, 2000 ▸). In addition, neutron and X-ray datasets are isomorphous ruling out any obvious unit-cell expansion during X-ray data collection, a global indicator of radiation damage (Garman, 2010 ▸).

As typically observed for protiated UOX crystals, the ^D^UOX–8AZA–W1 complex crystallizes in the orthorhombic space group *I*222. The asymmetric unit contains a single ^D^UOX chain that generates the biological tetramer of *D*2 symmetry following the application of crystallographic symmetry operations. Joint neutron/X-ray refinement delivered a final model characterized by *R*/*R*_free_ values of 21.47%/23.03% and 10.16%/10.93% for neutron and X-ray data, respectively. ^D^UOX residues are visible for residues 1–295, whereas the six C-terminal residues are flexible. In addition to the 8AZA ligand, 259 solvent molecules are modelled as D_2_O and 92 additional ones are modelled as oxygen atoms only owing to rotational disorder. Refinement statistics are summarized in Table 2 ▸. The use of perdeuterated UOX results in nuclear scattering length density free from protium-induced cancellation effects which allows the visualization of both exchangeable and non-exchangeable hydrogens. Representative examples of the excellent quality of X-ray and neutron maps are given in Figs. 2 ▸(*d*), 2(*e*) and S2.

A superposition of the final room-temperature ^D^UOX–8AZA–W1 model with that of the 1.75 Å resolution UOX–8AZA–W1 X-ray structure solved at 4°C (277 K) (PDB entry 1r51; Retailleau *et al.*, 2004 ▸) or with that of H/D-exchanged 1.1 Å resolution ^H/D^UOX–8AZA–Cl^−^ X-ray structure solved at 100 K (PDB entry 4n9v; Oksanen *et al.*, 2014 ▸) reveals essentially no differences, with RMSD values of 0.18 and 0.22 Å, respectively, for all common main-chain atoms. Thus, in line with what has been reported for other proteins (Koruza *et al.*, 2019 ▸), partial or complete deuterium-labelling does not induce major structural changes in UOX.

### 3.3. AZA and W1   

**Figure 3:**
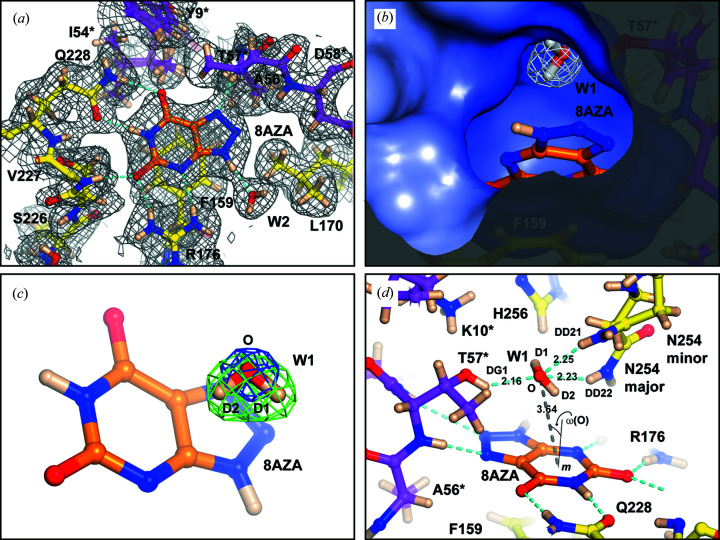
8AZA and W1. (*a*) 8AZA monoanion (orange) bound in the active site viewed from the peroxo hole. 2*mF*_o_ − *DF*_c_ neutron density map is shown in grey at the 1.0σ level for active site residues. Amino acids shown in yellow and magenta belong to different UOX protomers. Nitro­gen, oxygen and deuterium atoms are shown in blue, red and wheat, respectively. Hydrogen bonds are shown as cyan broken lines. Residues labelled with an asterisk indicate that they belong to a different UOX protomer. (*b*) Sliced-surface view of W1 bound in the peroxo hole located above the flat binding pocket occupied by 8AZA. The molecular surface is coloured according to the electrostatic potential with positive potential shown as shades of blue. 2*mF*_o_ − *DF*_c_ neutron density map for W1 is shown at the 1.5σ level. (*c*) W1 is present as a neutral H_2_O molecule. 2*mF*_o_ − *DF*_c_ electron density map shown in blue at the 3.0σ level clearly define the W1^O^ position. An omit *mF*_o_ − *DF*_c_ neutron map calculated only with W1^O^ contribution indicates the presence of two deuterons (D1, D2) as suggested by the elongated positive density (in green at the +3.0σ level) next to the oxygen atom. (*d*) W1 is sandwiched between Thr57* and Asn254. The latter is modelled in two conformations that refine at 0.8 (major) and 0.2 (minor) occupancy, respectively. W1 is oriented such that its D2 atom forms an O—H⋯π hydrogen bond with 8AZA. Useful parameters to describe this type of interaction are the O–*m* distance (broken grey line), where *m* is the midpoint of the six-membered pyrimidine ring, the ω(O) angle between the O⋯*m* line and the ring normal (thin continuous black line), as well as the O—D2⋯*m* angle (Steiner & Koellner, 2001 ▸). These values are 3.64 Å, 23.8° and 141°, respectively.

As observed in previous UOX complexes (Colloc’h *et al.*, 1997 ▸, 2007 ▸; Retailleau *et al.*, 2004 ▸; Oksanen *et al.*, 2014 ▸), 8AZA binds in the active site at the interface between two UOX protomers with direct hydrogen-bond stabilizing contributions from Thr57*, Asp58*, Arg176, Val227 and Gln228, while residues Tyr8*, Ile54*, Ala56*, Phe159, Leu170 and Ser226 further line the binding pocket with Phe159 engaged in π–π stacking with the pyrimidine ring of 8AZA [Fig. 3 ▸(*a*)]. We observe dual conformations for the side chains of Tyr8* and Ile54*, as well as for Ile288 located directly above Gln228.

8AZA binds as the N3 monoanion with clear nuclear scattering length density for deuterons linked to N1 and N9. Although nuclear scattering length density in omit maps appeared somewhat stronger for the hydrogen bound to N1, occupancy refinement gives unitary values for both deuterons. A water molecule (W2) forms a strong hydrogen bond with 8AZA^D9^ (8AZA^D9^⋯W2^O^ = 1.84 Å), though there is no evidence for a solvent molecule interacting with 8AZA^N8^ as seen in the anaerobic complexes with UA and its 9-methyl derivative (9-MUA), both of which can react with O_2_ to form C5-peroxides (Bui *et al.*, 2014 ▸).

W1 is visualized in the electrostatically positive peroxo hole above the 8AZA plane and with its oxygen atom facing Thr57* [Fig. 3 ▸(*b*)]. Electron density maps clearly define the position of the W1 oxygen (W1^O^), whereas omit neutron maps calculated by modelling W1 as W1^O^ only show elongated positive difference density next to the oxygen, which is consistent with the presence of two deuterium atoms [Fig. 3 ▸(*c*)]. In the final model W1 refines to an overall occupancy of 0.93. It is sandwiched between Thr57* and Asn254 with its plane oriented at ∼45° with respect to that of 8AZA with its O—D1 bond almost parallel to it and directed into a small cavity lined by 8AZA, Lys10*, Thr57*, Asn254 and His256 [Fig. 3 ▸(*d*)]. W1^O^ is hydrogen bonded to Thr57*^DG1^ at a distance of 2.16 Å. Similar distances (2.23–2.25 Å) are also observed between W1^O^ and the amide deuterons of Asn254, which, similar to other residues lining the active site (Tyr8*, Ile54*, Ile288), also exhibit alternative conformations. The visualization of W1 hydrogens allows us to also define an unanticipated O—H⋯π interaction with the ligand. Following the classical parametrization for this type of bond (Steiner & Koellner, 2001 ▸), which uses the distance (*d*_O⋯*m*_) between the donor atom (O) and the midpoint of the ring (*m*), the ω(O) angle between the O⋯*m* line and the ring normal as well as the O—H⋯*m* angle [Fig. 3 ▸(*d*)], we find values of *d*_O⋯*m*_ = 3.64 Å, ω(O) = 23.8° and ∠(O—H⋯*m*) = 141° that are consistent with those most commonly observed for the peptide *n* → (*n* − 2) N—H⋯π interaction for which there are abundant data in proteins (Steiner & Koellner, 2001 ▸).

### 3.4. Lys10*–Thr57* dyad   

**Figure 4:**
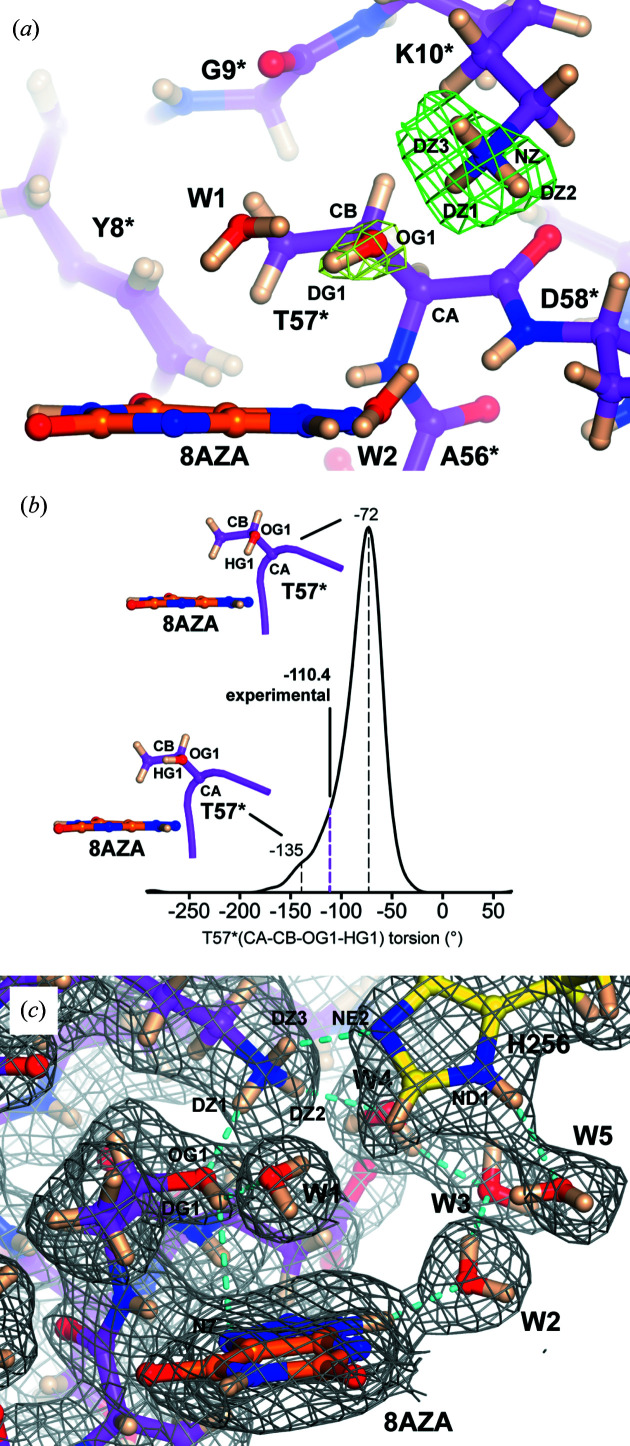
Lys10*–Thr57* dyad. (*a*) Stick representation of a portion of the active site highlighting the Lys10*–Thr57* dyad. Difference neutron density calculated in the absence of deuterons bound to Lys10*^NZ^ and Thr57*^OG1^ is shown at the +3.0σ (green) and +2.0σ (lemon) levels, respectively. No significant peak was observed near Thr57*^OG1^ at the +3.0σ level. (*b*) Normalized probability distribution of the Thr57* (CA–CB–OG1–HG1) torsion angle during a 100 ns classical MD simulation. Stick representations highlight the structures at −72° (OG1–HG1 bond pointing toward the triazole ring of 8AZA) and −135° angles (OG1–HG1 bond parallel to the 8AZA plane). The broken magenta line at −110.4° indicates the torsion angle in the final structure. (*c*) Protonation state in the active site. A strong hydrogen bond is formed between Lys10*^DZ1^ and Thr57*^OG1^ (1.97 Å) while Thr57*^DG1^ is engaged in an O—H⋯π interaction (2.77 Å) with N7 of the 8AZA triazole ring. A chain of water molecules (W2, W3, W4) connects 8AZA to the dyad. His256 is neutral with its hydrogen located on ND1 which is hydrogen bonded to W5. Cyan broken lines represent hydrogen bonds. Residue Asn254 has been omitted for clarity.

The active-site dyad formed by Lys10*–Thr57* has been implicated in various stages of the catalytic cycle (Gabison *et al.*, 2010 ▸; Imhoff *et al.*, 2003 ▸; Wei *et al.*, 2017 ▸), thus its proton­ation state was carefully investigated. While difference neutron maps for Lys10* showed a clear ‘tri-lobe’ nuclear density distribution around NZ that is consistent with a positively charged (Lys10*–ND_3_^+^) group [Fig. 4 ▸(*a*)], the identification of the protonation state for Thr57* was not so straightforward. Difference neutron maps did not provide evidence for a positive peak adjacent to Thr57*^OG1^ at the +3.0σ level but positive density that can be attributed to DG1 was seen at the +2.0σ level [Fig. 4 ▸(*a*)]. As neutron omit maps offered only a mild suggestion for the presence of a deuteron bound to Thr57*^OG1^ and difference map interpretation at low threshold levels need to be considered very carefully, we decided to investigate the unlikely possibility of a Lys10*–NH_3_^+^⋯O^−^–Thr57* ionic pair using theoretical methods. However, this scenario was quickly ruled out as already during the first few frames of QM/MM minimization (order of a few femtoseconds) a spontaneous proton transfer occurs from the Lys10* amine to the Thr57* hy­droxy­late resulting in the establishment of a Lys10*–NH_2_⋯HO–Thr57* hydrogen bond (Fig. S3). This strongly argues against the deprotonation of Thr57*.

We then considered the possibility that Thr57* is in the neutral state and that the lack of clear density for DG1 in neutron difference maps can be attributed to positional heterogeneity which cannot be interpreted fully at the resolution of our neutron diffraction data (2.10 Å). To further explore this, we calculated the distribution of the CA–CB–OG1–HG1 torsion angle during a 100 ns classical MD simulation. As shown in Fig. 4 ▸(*b*), this dihedral exhibits a rather broad asymmetric profile ranging from −10 to −190° with a maximum at around −72°, resulting in the OG1–HG1 bond pointing ‘down’ towards 8AZA, almost perpendicular to it. A smaller ‘shoulder’ is also seen around −135°, with OG1–HG1 parallel to 8AZA. We therefore modelled a deuteron atom bound to Thr57*^OG1^ as suggested by omit neutron maps. Occupancy refinement for this atom gives a value of 0.78. In the final model the CA–CB–OG1–DG1 torsion angle displays a value of −110.4° resulting in a slight downward orientation of the OG1–DG1 bond towards the ligand plane with the deuteron at 2.77 Å from 8AZA^N7^, thus indicating another O—H⋯π interaction [Fig. 4 ▸(*c*)]. All deuterons bound to Lys10*^NZ^ also refine to high occupancy (>0.90) with a hydrogen bond between Lys10*^DZ1^ and Thr57*^OG1^ at a distance of 1.97 Å. Lys10* is in turn hydrogen bonded to His256. The latter is neutral, with its hydrogen residing on ND1. As a result, His256^NE2^ accepts a hydrogen bond from the Lys10*^DZ3^, positioned at a distance of 2.24 Å. Lys10*^DZ2^ is hydrogen bonded to water W4 that, together with W5 and W2, completes a chain of solvent molecules connecting the Lys10*–Thr57* dyad to AZA^D9^. Overall, experimental data and theoretical calculations are consistent with the existence of a Lys10*–ND_3_^+^⋯DO–Thr57* dyad in which the Thr57*^OG1–DG1^ bond is directed towards 8AZA, on average.

### 3.5. Heterogeneity of Asn254 and its flanking residues   

**Figure 5:**
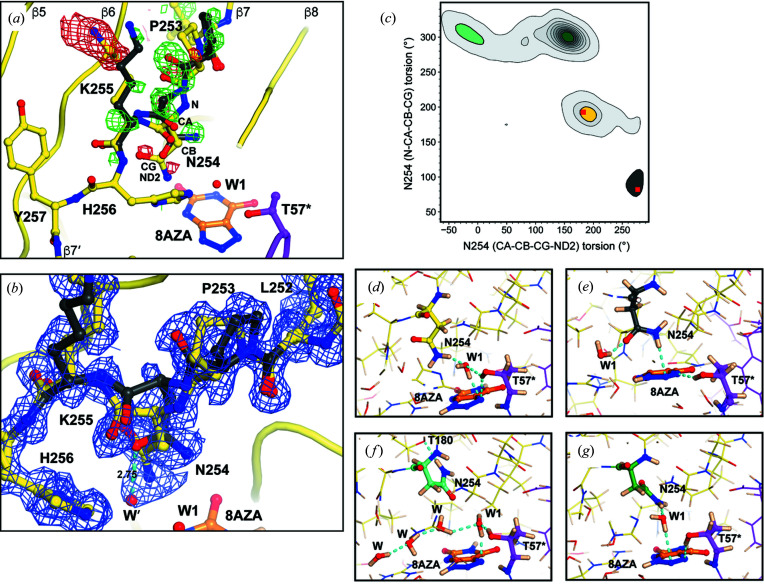
Asn254 heterogeneity. (*a*) Close-up of the β5–β8 region highlighting an alternative conformation for the Pro253–Lys255 tripeptide. Strands are shown as tubes and the stretch connecting β7 to β7′ as sticks. Deuterium atoms have been omitted for clarity. The main conformation for the Pro253–Lys255 tripeptide is shown in yellow and the second conformation is represented in dark grey for reference. Difference *mF*_o_ − *DF*_c_ electron density maps at the +3.0σ and −3.0σ levels calculated after refinement without the minor conformation included in the model are shown in green and red, respectively. The alternative conformation stretch explains the residual density well. (*b*) Feature-enhanced 2*mF*_o_ − *DF*_c_ map (FEM) for the Pro253–Lys255 tripeptide and neighbouring residues. Colour codes are the same as in (*a*). The solvent molecule W′ is hydrogen bonded to the oxygen atom of the Asn254 amide in the minor conformation. (*c*) 2D contour plot for Asn254 torsion angles from MD simulations highlighting four main clusters represented by different colours (green, yellow, aqua and dark grey). Red squares within the yellow and dark grey clusters identify experimental torsion angles for the major (179°, −192°) and minor (272°, 81°) Asn254 conformations, respectively. (*d*)–(*g*) Representative structures of each torsion angle cluster identified by the simulations: (*d*) yellow, (*e*) dark grey, (*f*) aqua, (*g*) green. 8AZA, Thr57*, Asn254, W1 and other water molecules at hydrogen-bond distance are shown as sticks. Asn254 is colour-coded according to the cluster to which it belongs. Hydrogen bonds are shown by broken cyan lines.

During crystallographic refinement, inspection of near-atomic resolution X-ray difference maps showed a pattern of positive and negative peaks for the tripeptide linker Pro253–Lys255 connecting the β7 and β7′ strands. This indicated the existence of an alternative conformation in addition to the main one typically observed, which we could model [Fig. 5 ▸(*a*)]. Occupancy refinement for this short stretch that hosts Asn254 suggests an approximate 75:25 ratio for the major [yellow in Fig. 5 ▸(*a*)] and minor (dark grey) conformations. In the latter, Pro253 is shifted towards β8 resulting in a movement of Asn254 in the opposite direction. This leads to an Asn254^CA^ displacement of ∼1.4 Å and the consequent movement of its side chain that nevertheless remains at hydrogen-bond distance from W1. Modelling of this region in an alternative conformation made a residual peak more obvious in *mF*_o_−*DF*_c_ electron density maps at the +4.0σ level (∼0.25 e^−^ Å^−3^) near the amide group of Asn254 in its major conformation (Fig. S4). We interpreted this peak as a low-occupancy water molecule (oxygen atom only, refined occupancy = 0.21). Inclusion of this solvent molecule in the model fully accounted for the positive difference density. Moreover, we calculated 2*mF*_o_−*DF*_c_ feature enhanced maps (FEM) that can strengthen weak signals, if present, and can reduce model bias and noise (Afonine *et al.*, 2015 ▸). The FEM map for this region shows clearly the alternative conformation for Pro253–Lys255 peptide as well as a ‘bulge’ on the side of the amide plane that supports our modelling of this low-occupancy water (W′) [Fig. 5 ▸(*b*)].

To further validate our experimental observation of flexibility for this region we looked at the distribution of Asn254 torsion angles using MD simulations [Fig. 5 ▸(*c*)]. Two independent 100 ns simulations were carried out using a UOX dimer model (thus allowing the analysis of two active sites in each run) (Fig. S5) with the Pro253–Lys255 stretch either in the major or minor conformation. The initial 25 ns of each simulation were removed from the analysis to avoid bias caused by system conditioning. As shown in Fig. 5 ▸(*c*), the MD simulations allow us to define four main cluster areas (green, yellow, aqua, dark grey) in the (CA–CB–CG–ND2)–(N–CA–CB–CG) 2D contour plot. The cluster highlighted in yellow is representative of the main conformation seen in the crystal [red square at 179°, 192° in Fig. 5 ▸(*c*)] and confirms that this rotamer is sampled often during the simulation. A snapshot from the simulation illustrative of this cluster is given in Fig. 5 ▸(*d*) and shows an arrangement similar to that seen in the crystal. The dark grey cluster representing the minor crystallographic conformation (red square at 272°, 81°) is also occasionally visited, thus lending support to our structural observation. In many structures of this cluster W1 moves away from the peroxo hole with the hydrogen of the Asn254 amide replacing W1 in an N—H⋯π interaction with 8AZA [Fig. 5 ▸(*e*)]. This novel W1 position appears consistent with that of that of the low-occupancy W′ in our model.

The simulations also reveal additional torsion angles frequently adopted by Asn254 that define almost a continuum (aqua and green clusters). Representative structures are shown in Figs. 5 ▸(*f*), and 5(*g*), respectively. Structures belonging to the aqua cluster display the Asn254 side-chain carbonyl group pointing towards the peroxo hole typically resulting in the displacement of W1 and the establishment of a solvent chain organized above the ligand [Fig. 5 ▸(*f*)]. The transition from the aqua to the green cluster involves a 90° rotation of the amide plane resulting in the carbonyl ‘pointing away’ from the peroxo hole and thus establishing a hydrogen bond between one of the HD hydrogens and W1 [Fig. 4 ▸(*g*)]. Taken together, experiment and theory indicate that Asn254 is rather dynamic and able to explore a range of conformations.

### 3.6. Asn254–W1 coupling   

**Figure 6:**
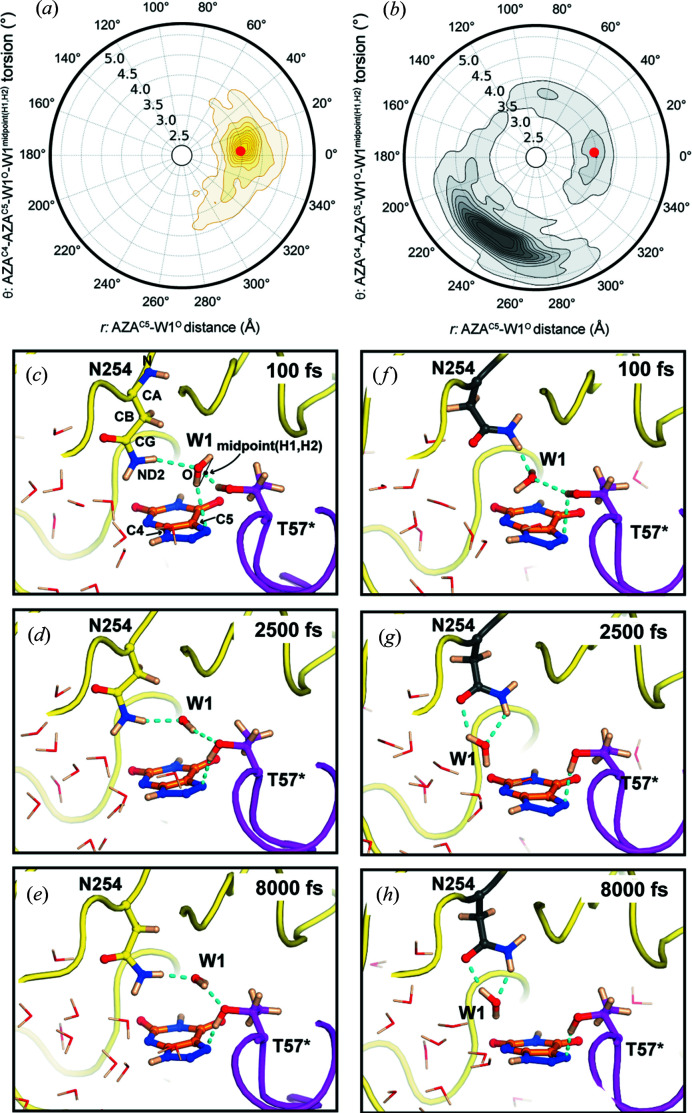
The W1 position is coupled to Asn254 conformations. (*a*) Radial (*r*, θ) plot for the QM/MM-MD simulation with Asn254 in the major conformation. The distance AZA^C5^–W1^O^ is shown along *r* with the AZA^C4^–AZA^C5^–W1^O^–W1^midpoint(H1,H2)^ torsion angle as θ. The red circle indicates values for these parameters derived from the crystal structure. W1 is relatively stable during the simulation at values close to experimental ones. (*b*) Following (*a*), but for the QM/MM-MD simulation with Asn254 in the minor conformation. In contrast to what is shown in (*a*), when Asn254 is in the minor conformation W1 moves to greater distances from C5 (maximum at 4.2 Å) exhibiting a broader distribution of different (225–255°) dihedral angles. (*c*)–(*h*) Cartoon snapshots from the simulations. (*c*)–(*e*) are from the simulation starting with the Pro253–Lys255 region in the major crystallographic conformation; (*f*)–(*h*) are from the simulation starting with the Pro253–Lys255 region in the minor crystallographic conformation. Thr57*, Asn254, W1 and 8AZA are in stick form with the protein backbone shown as a tube. Other water molecules are shown as lines. Broken cyan lines indicate hydrogen bonds. The minor Asn254 conformation facilitates W1 removal from the peroxo hole by stabilizing it in a new position toward the solvent-exposed of the active site at longer distance from C5. Supplementary Movies S1 and S2 are provided for the QM/MM-MD simulations.

Together with Thr57*, Asn254 is involved in W1 binding and also in the stabilization of O_2_ and 5PIU during the oxidative stage of catalysis (Bui *et al.*, 2014 ▸). As both H_2_O and O_2_ must access the peroxo hole during the reaction cycle we considered the possiblity that Asn254 dynamics might offer a mechanism to modulate W1 stability at this location. To investigate this further, we performed QM/MM MD simulations. Similar to the approach employed in our classical MD simulations, we performed independent 8 ps-long runs using initial structures in which the Pro253–Lys255 region was either in the major or minor conformation. Asn254 torsion angles remain stable during the simulations indicating energetic minima (Fig. S6). We then inspected W1 movements using the AZA^C5^–W1^O^ distance and the AZA^C4^–AZA^C5^–W1^O^–W1^average (H1,H2)^ dihedral angle as coordinate parameters. For the major conformation [Fig. 6 ▸(*a*)], W1 mostly explores distances between 3.1 and 4.0 Å with a maximum at 3.6 Å and a narrow dihedral distribution centred around 10°. This is in excellent agreement with the experimental values in our structure (3.47 Å, 6°). The snapshots at different time points in Figs. 6 ▸(*c*), 6(*d*) and 6(*e*) provide further visual confirmation that W1 is relatively stable within the peroxo hole when Asn254 is in the major conformation (see Supplementary Movie S1 in the supporting information). The situation is different for the minor conformation [Fig. 6 ▸(*b*)]. Under these conditions W1 moves at longer distances from AZA^C5^ (maximum at 4.2–4.4 Å) exhibiting a broader distribution of different dihedral angles (225–255°). This is the result of W1 leaving the peroxo hole (we observe this after ∼500 fs) stabilized by the amide group of Asn254 as seen in the snapshots in Figs. 6 ▸(*f*) 6(*g*) and 6(*h*) (see Supplementary Movie S2). In our final crystallographic model W′ refines at a distance of 4.4 Å from AZA^C5^ and at hydrogen-bonding distance of 2.75 Å from Asn254^OD1^ (minor). Taken together, experiment and theory (both standard MD and QM/MM-MD) are in excellent agreement and support the view that the minor crystallographic conformation favours the displacement of W1 from the peroxo hole.

## 4. Discussion   

The synergistic use of experimental and theor­etical approaches provides a powerful way to gain insight into the mechanisms of enzyme catalysis (Bui & Steiner, 2016 ▸; Huggins *et al.*, 2019 ▸). Here, we have integrated high-resolution room-temperature neutron/X-ray crystallographic analysis with biomolecular simulations to study co-factor-independent UOX in complex with its competitive inhibitor 8AZA in the presence of the catalytic water molecule W1 bound in the peroxo hole.

Our room-temperature analysis has uncovered structural heterogeneity for the Pro253–Lys255 tripeptide. This involves residue Asn254 which interacts directly with W1. High-resolution crystallographic studies at room temperature are extremely valuable in the detection of functional multiconformers or rare states in enzymes restricted under cryogenic conditions (Fraser *et al.*, 2011 ▸). Early studies involving crystals of myoglobin (Frauenfelder *et al.*, 1987 ▸, 1979 ▸) and ribonuclease (Rasmussen *et al.*, 1992 ▸; Tilton *et al.*, 1992 ▸) have shown that conformational distributions are restricted at low temperatures and, for human proline isomerase cyclo­philin A, only collection of X-ray diffraction data at ambient temperature allowed an agreement between the crystallographic and NMR functional conformational substates to be obtained (Fraser *et al.*, 2009 ▸). The two Asn254 conformers observed here map onto high-probability clusters of torsion-angle distributions obtained from the simulations [yellow and dark grey clusters in Fig. 5 ▸(*b*)]. Asn254 side-chain variability has also been observed under ligand-free conditions (Retailleau *et al.*, 2004 ▸). In the absence of a ligand, UOX has been crystallized in a different space group (*P*2_1_) with two complete tetramers in the asymmetric unit (PDB entry 1r56; Retailleau *et al.*, 2004 ▸). In the 2.6 Å resolution structure at 288 K, Asn254 samples a range of states that map onto the aqua–green clusters in Fig. 5 ▸(*b*). Conformational heterogeneity for Asn254 therefore appears to be a constitutive property of UOX. In cholesterol oxidase flavoenzyme, an arginine and a glutamate residue at the active site, found in two conformations, have been proposed to control oxygen access to the active-site cavity from a neighbouring channel (Coulombe *et al.*, 2001 ▸). It is tempting to suggest that Asn254 dynamics might play a similar role considering that this residue marks the boundary between the active site and an adjacent hydro­phobic cavity proposed to act as a transient O_2_ reservoir (Colloc’h & Prangé, 2014 ▸).

A mechanism for O_2_ gating facilitated by Asn254 dynamics probably involves W1 destabilization. MD simulations show that, though the main crystallographic conformation favours long W1 residence times in the peroxo hole, the minor conformation is associated with a higher probability of escaping from it. In fact, this conformation stabilizes W1 outside the peroxo hole at a position consistent with that of the low-occupancy W′ in our structure. It is therefore possible that the marginally lower than unitary occupancy for W1 (occupancy 0.93) arises from its fractional mobilization to the W′ site promoted by the minor conformation of Asn254. Coupling of Asn254 conformational heterogeneity and W1 stability is further supported by the crystal structure of ligand-free UOX in which Asn254 is visualized adopting multiple alternative rotamers (Retailleau *et al.*, 2004 ▸). Here, W1 is observed in only three of the eight independent active sites of the two UOX tetramers present in the asymmetric unit. A mechanism that takes advantage of protein dynamics to ‘actively’ promote W1/O_2_ exchange for the oxidation step of catalysis appears particularly useful for cofactor-independent UOX as O_2_ affinity for the electrostatically positive active site is presumably rather low. Therefore, similarly to other cofactor-independent O_2_-metabolizing enzymes such as DpgC, poor ability to concentrate dioxygen in the active site, for example by metal coordination, must be compensated by mechanisms that increase the frequency of O_2_ entries (Di Russo *et al.*, 2015 ▸). Asn254 dynamics is therefore suggested to decrease W1 residence times favouring O_2_ exchange with the neighbouring hydro­phobic pocket.

The availability of the joint neutron/X-ray structures of ^D^UOX–8AZA–W1 allows for an interesting comparison with that of the UOX–8AZA–Cl^−^ complex (PDB entry 4n3m) solved in previous work (Oksanen *et al.*, 2014 ▸). Functionally, the key difference between these structures resides in the nature of the molecule present in the peroxo hole (water versus chloride). Thus, comparative analysis is particularly meaningful for active-site residues. While both structures show that 8AZA is bound as a monoanion deprotonated at N3, differences exist for the important Lys10*–Thr57* dyad (Fig. S7). In UOX–8AZA–Cl^−^, Lys10* is modelled in the neutral NH_2_ state accepting a hydrogen bond from Thr57* whose DG1 atom points toward Lys10*^NZ^. The authors, however, state that a second conformation (not modelled) might exist in which Lys10* is positively charged (–NH_3_^+^) and the protonated Thr57* hydroxyl group points towards the Cl^−^ ion. In UOX–8AZA–W1, the catalytic water W1 is present in the peroxo hole as a neutral H_2_O (here we will use H for both ^1^H and D) oriented at 45° with respect to the ligand plane and stabilized by a combination of hydrogen bonds. These involve not only the side chains of Thr57* and Asn254 (both conformations) but also an O—H⋯π interaction with the 8AZA monoanion. We find clear evidence for a charged Lys10*–NH_3_^+^ side chain whilst the Thr57*^HG1^ atom does not appear to be strongly localized. However, our experiment and simulation agree that the Thr57*^OG1–HG1^ bond points, on average, in the direction of the ligand, *i.e.* rotated by almost 180° compared with what was reported in the presence of chloride. We suggest that differences in active-site protonation states between these structures most likely reflect the nature and charge of the different molecules bound in the peroxo hole. In future work it will therefore be important to determine the neutron structure of the UOX–UA–W1 complex under anaerobic conditions as this has direct implications on the mechanism of substrate-induced O_2_ activation.

**Figure 7:**
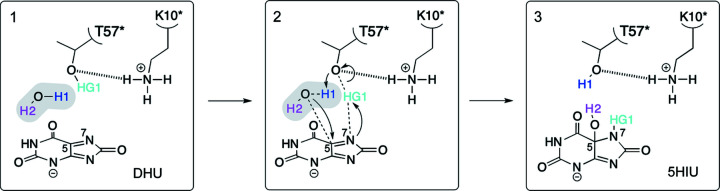
Hydration step. Reaction mechanism for the hydration step in UOX catalysis.

The protonation state observed in our UOX–8AZA–W1 complex is directly relevant for the hydration stage of the reaction mechanism. A recent theoretical study (Wei *et al.*, 2017 ▸) proposed the existence of a strong hydrogen-bonding network between charged Lys10*, Thr57*, W1 and DHU^N7^ (note that in their paper the authors use protein numbering for *B. subtilis* UOX in which Lys9* and Thr69* correspond to *A. flavus* Lys10* and Thr57*, respectively). As W1^O^ approaches DHU^C5^ in the nucleophilic attack, W1^H1^ comes close to Thr57*^OG1^ while HG1 gradually forms a bond with DHU^N7^ changing the C5=N7 double bond to a single bond (Fig. 7 ▸). In this mechanism the positively charged Lys10* stabilizes the negative charge on Thr57* during the transition state. Our structural data and simulation results are fully consistent with this proposal. Specifically, the charged Lys10* side chain favours the orientation of the Thr57*^OG1–HG1^ bond toward the π system of the ligand, thus facilitating proton transfer to N7. As W1^O^ is oriented toward C5, nucleophilic attack is expected to occur on this atom (instead of C4) while Thr57* regains neutrality by abstracting the H1 proton from W1. Thr57^HG1^ is therefore central to a Lys10–NH_3_^+^-assisted proton-relay system.

## Supplementary Material

Supporting movie 1. DOI: 10.1107/S2052252520013615/jt5052sup1.mpg

Supplorting movie 2. DOI: 10.1107/S2052252520013615/jt5052sup2.mpg

Supporting figures. DOI: 10.1107/S2052252520013615/jt5052sup3.pdf

PDB reference: perdeuterated urate oxidase from *Aspergillus flavus* in complex with 8-azaxanthine, 7a0l

## Figures and Tables

**Table 1:** Neutron and X-ray data collection statistics

Dataset	^D^UOX–8AZA–W1 neutron	^D^UOX–8AZA–W1 X-ray
Beamline	LADI-III, ILL	BM30A, ESRF
Temperature (K)	293	293
Wavelength (Å)	3.12–4.20	0.9800
Exposure time per image	1.5 h	5 s
Number of images	11	240
Oscillation angle (°)	10	1
Resolution range† (Å)	40.00–2.10 (2.21–2.10)	52.70–1.33 (1.35–1.33)
Space group	*I*222	*I*222
Cell dimensions *a*, *b*, *c* (Å)	80.63, 96.13, 105.40	80.63, 96.13, 105.40
Unique reflections†	19234 (2357)	93643 (4505)
Overall redundancy†	3.4 (3.3)	9.2 (5.3)
Completeness† (%)	80.2 (68.1)	99.8 (96.9)
*R*_merge_ † (%)	14.1 (38.3)	4.1 (127.3)
*R*_p.i.m._ (*I*)† (%)	7.9 (21.5)	1.4 (59.8)
〈*I*/σ(*I*) 〉†	5.8 (2.9)	24.4 (1.2)
CC_1/2_ † (%)	98.7 (82.6)	100.0 (46.5)

† Numbers in parentheses refer to the highest resolution bin.

**Table 2:** Joint neutron/X-ray refinement statistics

PDB code	7a0l
Neutron *R*_factor_/*R*_free_ (%)	21.47/23.03
X-ray *R*_factor_/*R*_free_ (%)	10.16/10.93
Number of protein residues, solvent molecules, ligands, ions	295, 351 (259 as D_2_O; 92 as O only), 1 (8AZA), 1 (Na^+^)
Average *B* value (Å^2^) protein, solvent, 8AZA, Na^+^	24.1, 45.4, 15.3, 25.5
R.m.s. bond lengths (Å)	0.010
R.m.s. bond angles (°)	1.411
