# Supplementary material for: Joint neutron/X-ray crystal structure of a mechanistically relevant complex of perdeuterated urate oxidase and simulations provide insight into the hydration step of catalysis
Source: IUCrJ. 2021 Jan 1;8(Pt 1):46–59. doi: 10.1107/S2052252520013615 (PMC7792999; doi:10.1107/S2052252520013615)
Supplement: Supplementary file 3 [file m-08-00046-sup3.pdf]

IUCrJ

Volume 8 (2021)

Supporting information for article:

Joint neutron/X-ray crystal structure of a mechanistically relevant complex of perdeuterated urate oxidase and simulations provide insight into the hydration step of catalysis

Lindsay McGregor, Tamas Foldes, Soi Bui, Martine Moulin, Nicolas Coquelle, Matthew P. Blakeley, Edina Rosta and Roberto A. Steiner

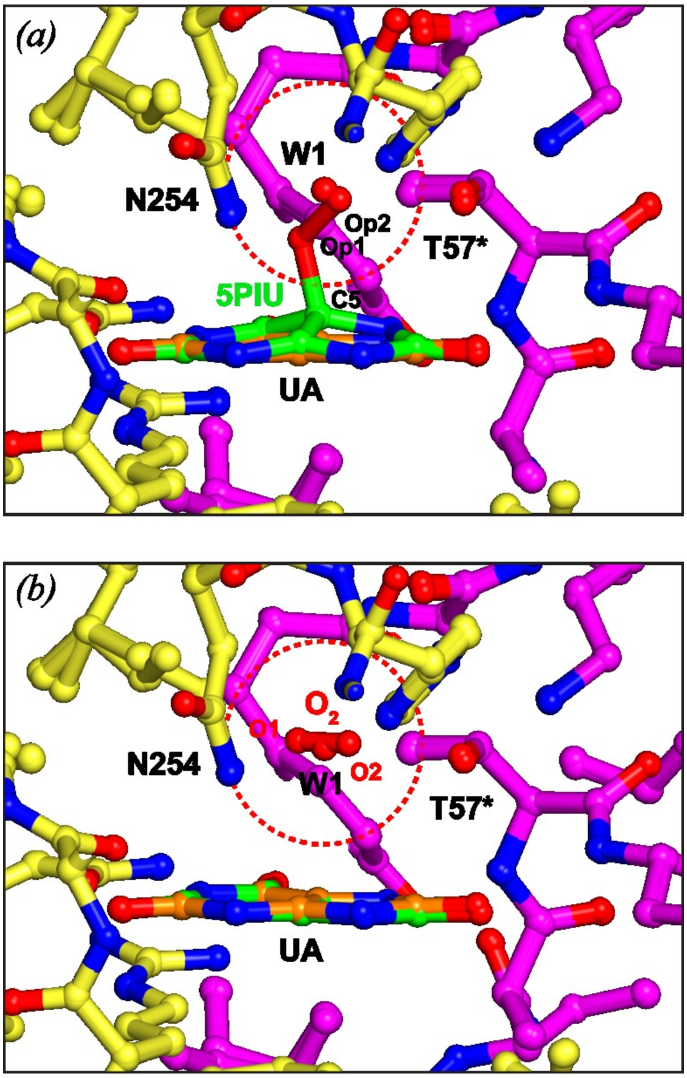

Figure S1 **Peroxo hole.** (*a*) Cartoon representation of the active site of the anaerobic UOX-UA-W1 complex (PDB code 4D12) superposed to that of the UOX-5PIU complex (PDB code 4D13). W1 occupies the same position of the Op2 atom of the trapped 5PIU peroxide intermediate; (*b*) Active site of the anaerobic UOX-UA-W1 complex (PDB code 4D12) superposed to that of the UOX-UA-O_2 complex (PDB code 4D19) trapped under cryo-conditions following selective radiolysis of the $5PIU^{C5-Op1}$ bond (Bui *et al.*, 2014). W1 occupies the midpoint of the diatomic O_2 molecule. In both panels the "peroxo hole" is highlighted by the broken red circle.

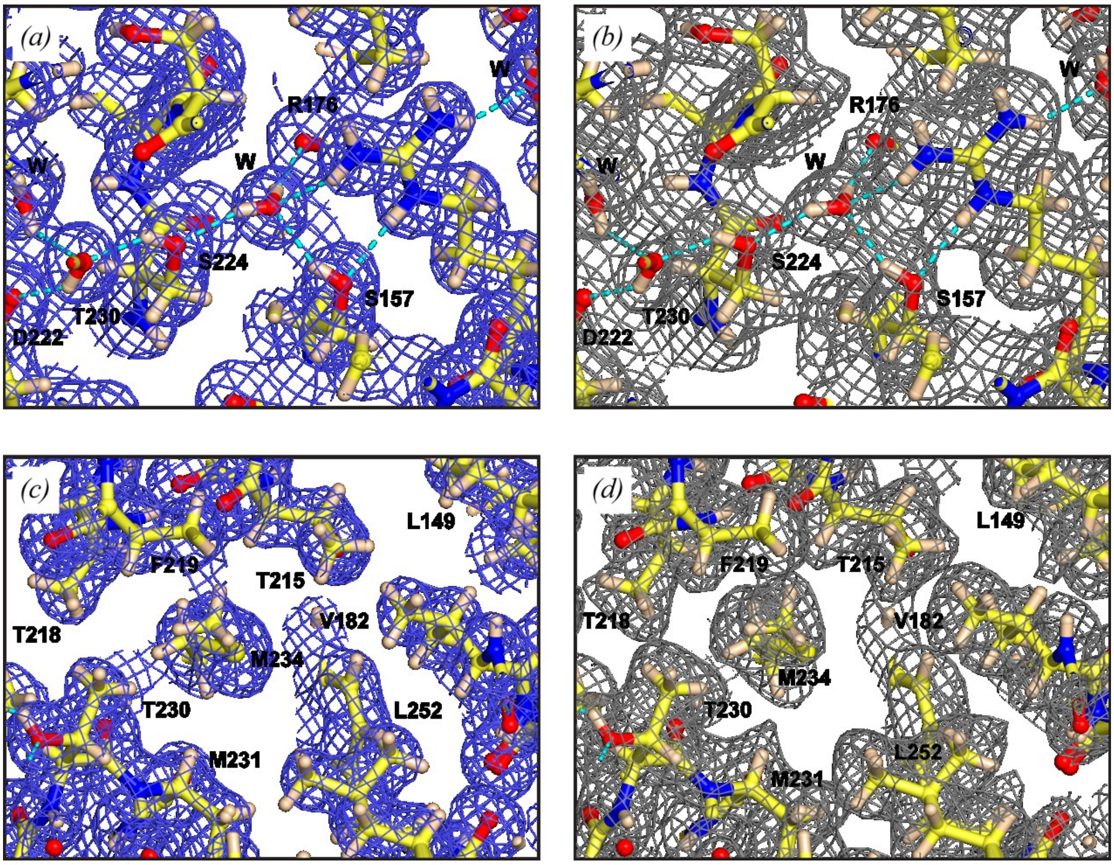

Figure S2 **Representative 2*mF*$_o$-*DF*$_c$ electron and neutron maps for the ^{D}UOX-8AZA-W1 complex.** (*a*,*c*) Electron density maps shown in blue at the 1σ level; (*b*,*d*) Neutron density maps shown in grey at the 1σ level for the same region as (*a*,*c*), respectively.

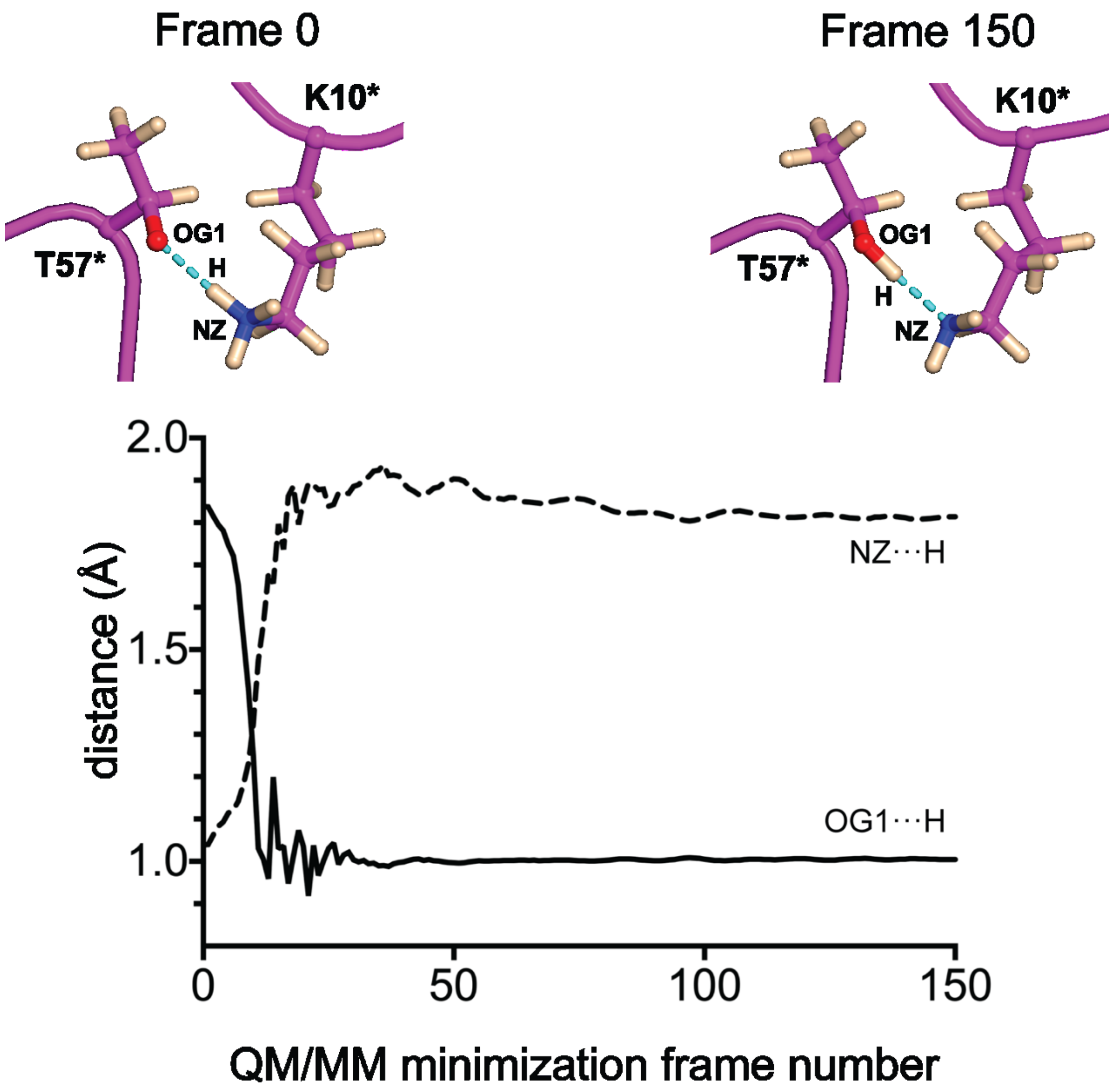

Figure S3 QM/MM calculations do not support the existence of a K10*-NH$_3^+$---O$^-$-T57* ionic pair. Proton (H) shifts from the K10* amine to the T57* hydroxylate during the initial stages of QM/MM minimization as indicated by changes in K10*^{NZ}···H and T57*^{OG1}···H distance. Cartoons show structures for the dyad at the beginning of the minimization (frame 0) and once the system is stable (frame 150).

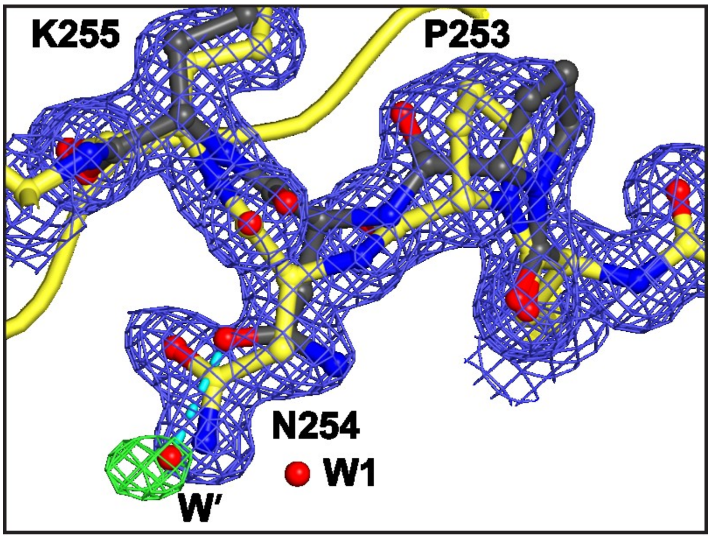

Figure S4 **Density supporting the modelling of the water molecule (W`).** $2mF_o$-$DF_c$ (blue) and $mF_o$-$DF_c$ (green) electron density maps contoured at the 0.8σ and +3.0σ level, respectively, for the P253-K255 stretch and flanking residues. Maps were calculated prior to the inclusion of the W` solvent molecule. The final model is shown as reference with the major (occupancy = 0.76) and minor (occupancy = 0.24) conformations for this tripeptide in yellow and dark grey, respectively. Solvent molecules are shown as red spheres. Deuterons are not shown for clarity. W` is H-bonded (cyan broken line) to the minor conformation of N254.

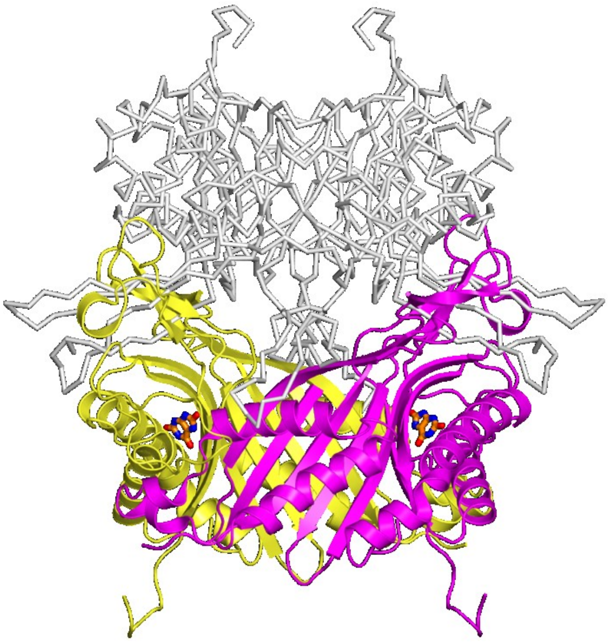

Figure S5 **UOX dimer for MD simulations.** Classical MD simulations were performed using a model that corresponds to the UOX dimer highlighted by the yellow and magenta protomers. This model encompasses two complete active sites hosting the 8AZA ligands shown in orange as stick representation.

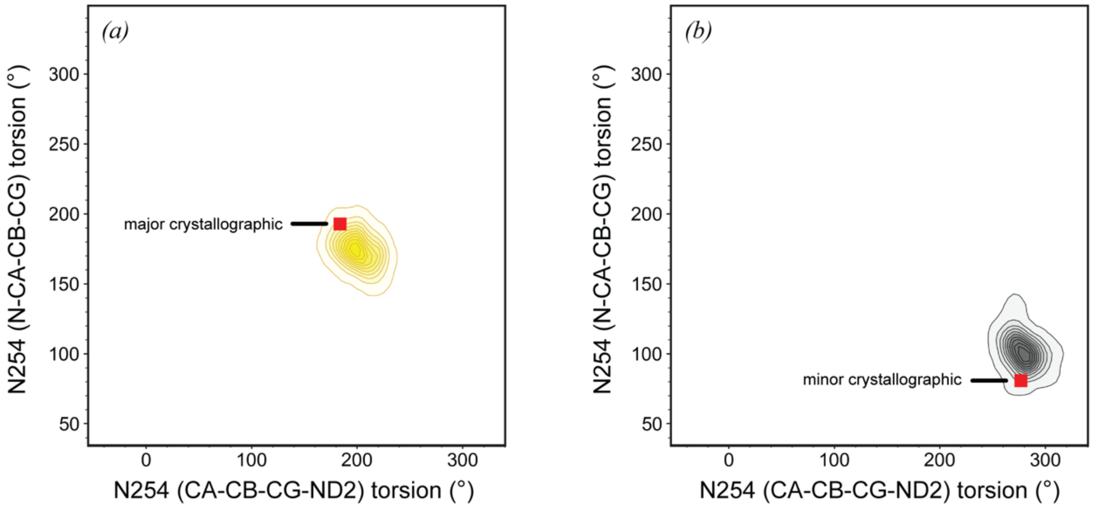

Figure S6 **N254 torsion angles during QM/MM-MD simulations.** (*a*) 2D contour plot of the (CA-CB-CG-ND2)/(N-CA-CB-CG) torsion angle distribution during the QM/MM-MD simulation in which the P253-K255 tripeptide is modelled in the major crystallographic conformation. The red square identifies experimental values; (*b*) As (*a*) but for the P253-K255 tripeptide in the minor crystallographic conformation. Torsion angles sampled during the QM/MM-MD simulations display a narrow distribution consistent with the experimental values indicating that the conformations seen in the crystal correspond to energetic minima.

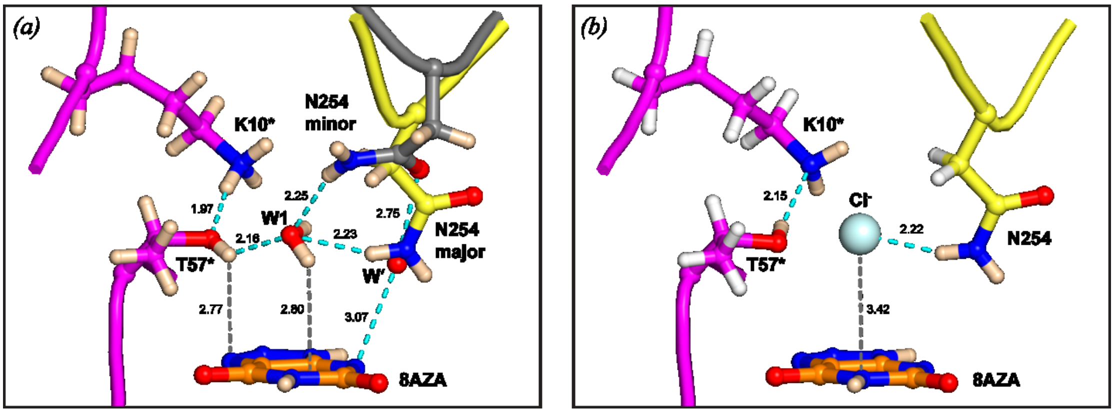

Figure S7. Comparison between active sites. (*a*) ^{D}UOX-8AZA-W1 (this work); (*b*) $^{H/D}$UOX-8AZA-Cl^{-} (PDB code 4N3M) (Oksanen *et al.*, 2014). H-bonds are indicated by broken cyan lines while interactions with the π system of 8AZA are in grey. D and H atoms are shown in wheat and white, respectively. Distances in Å are given.
